# New potential for an old kid on the block: Impact of premorbid metformin use on lactate kinetics, kidney injury and mortality in sepsis and septic shock, an observational study

**DOI:** 10.1002/edm2.382

**Published:** 2022-11-28

**Authors:** Nina Van Moorter, Thomas Tackaert, Koen De Decker, Bruno Van Vlem, Nikolaas De Neve

**Affiliations:** ^1^ Department of Internal Medicine Ghent University/Ghent University Hospital Ghent Belgium; ^2^ Department of Emergency Medicine Ghent University/Ghent University Hospital Ghent Belgium; ^3^ Department of Anaesthesiology and Critical Care Medicine OLVZ Aalst Aalst Belgium; ^4^ Department of Nephrology OLVZ Aalst Aalst Belgium

**Keywords:** acute kidney injury, lactate, lactate kinetics, metformin, sepsis management, septic shock

## Abstract

**Introduction:**

Sepsis and septic shock cause significant mortality worldwide, with no targeted molecular therapies available. Metformin has pleomorphic effects that may be beneficial in sepsis, but at present, the impact of metformin exposure on sepsis remains controversial. Metformin might alter lactate metabolism, but little is known about its influence on lactate kinetics. We therefore investigated the impact of preadmission metformin use on lactate kinetics, acute kidney injury (AKI) and mortality in sepsis.

**Materials and Methods:**

We retrospectively analysed all ICU admissions with sepsis and septic shock between January 2013 and September 2020, identifying 77 users and 390 nonusers (subdivided in diabetics, *n* = 48 and nondiabetics, *n* = 342).

**Results:**

(Sub)groups did not differ in illness severity or sepsis aetiology. Admission lactate levels were similar, but evolution in lactate over the first 24 h showed a larger decrease in users vs nonusers (median − 53% vs. −36%, *p* = .010). No difference in AKI or renal replacement therapy was found. Mortality was lower in users vs nonusers in case of septic shock (21.9% (*n* = 7) vs. 42.7% (*n* = 61) for 90d mortality, *p* = .029, OR 0.38 [95% CI: 0.15–0.93]), but showed no significant differences in the combined sepsis and septic shock population.

**Conclusions:**

In our data, preadmission metformin use is associated with a significantly larger decrease in lactate after admission with sepsis or septic shock and with reduced mortality in septic shock. This underscores the need for further studies investigating the interplay between metformin, lactate and sepsis, thereby exploring the potential use of metformin or its pathways in sepsis.

## Graphical abstract

Metformin’s pleomorphic effects may be beneficial in sepsis and septic shock, but its impact remains controversial. Our study is the first to show a significant association between metformin and lactate kinetics in sepsis or septic shock, with a larger decrease in lactate after admission. Metformin use was also associated with reduced mortality in septic shock. Further studies are needed exploring the potential use of metformin or its pathways in sepsis.

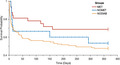

## 1 INTRODUCTION

Sepsis is a life‐threatening organ dysfunction caused by a dysregulated host response to infection, with in case of septic shock also underlying circulatory, cellular and metabolic abnormalities associated with a higher risk of mortality.^1^ It remains a significant healthcare problem, affecting millions of people around the world and causing significant attributed mortality,^2^, ^3^ despite substantially improved outcomes with improvements in supportive care.^2^, ^4^ Over the past decades, a large number of therapeutic agents have been evaluated in clinical trials without success.^2^, ^5^ Currently, there are no approved specific molecular therapies for sepsis.^2^, ^5^

Metformin, an oral antihyperglycaemic agent in the biguanide class, has become the drug of choice for treatment of diabetes mellitus type 2.^6^ The mechanisms by which metformin exerts its actions remain subject to much debate.^7^ Besides its glucose‐lowering effects, metformin has anti‐inflammatory and other pleiotropic effects that may be beneficial in sepsis or other critical illness.^8^, ^9^ In rodent models of sepsis, metformin treatment showed improved survival, protection against acute respiratory distress syndrome (ARDS) and sepsis‐induced organ failure and prevention of sepsis‐associated immunosuppression, possibly related to AMP‐activated protein kinase activation and IL‐6 and IL − 1 inhibition.^9^, ^10^, ^11^, ^12^ Metformin is a promising candidate host‐directed therapy for tuberculosis^13^ and increased the innate immune capacity to eradicate Pseudomonas aeruginosa in septic mice.^10^ In patients with COVID−19, metformin use was associated with mortality reduction beyond its effects on glycaemic control.^14^, ^15^ As such, metformin has been coined as a possible treatment for sepsis.^8^, ^9^

One of the difficulties of interpreting the effects of metformin in sepsis is the possible interference with the metabolism of lactate. Lactate is an important marker in sepsis and is independently associated with subsequent organ dysfunction and mortality, and part of the criteria for septic shock.^1^, ^16^, ^17^

Acute kidney injury (AKI) may on the one hand affect both clearance of metformin, leading to drug accumulation, and lactate.^18^, ^19^, ^20^, ^21^, ^22^ On the other hand, metformin may also positively affect renal function. It has been shown to protect against progression to end‐stage renal disease in diabetic patients with chronic kidney disease,^22^ but the effect of metformin on sepsis‐induced kidney injury remains controversial with variable outcomes being reported.^9^, ^23^, ^24^, ^25^, ^26^

We aimed at investigating the relation between premorbid metformin use, lactate and lactate kinetics in critically ill patients with sepsis or septic shock, by evaluating both static and dynamic lactate parameters. Until now, only one previous study reported dynamic lactate parameters in this subgroup of patients,^25^ although dynamic parameters are reported to have more predictive value.^27^, ^28^

Furthermore, this is to our knowledge the first study using both serum creatinine and diuresis data to analyse the association between metformin use and acute kidney injury (AKI) in this population over a timeframe of more than 24 h, and the second study to report having included urinary output data in general. Lastly, we also evaluated the association with both short‐ and longer term mortality.

## 2 MATERIALS AND METHODS

### 2.1 Setting and inclusion

We performed a retrospective cohort study, reviewing the electronic medical records of all patients admitted to the Intensive Care Unit (ICU) of the OLV hospital of Aalst, Belgium, between January 2013 and September 2020. The OLV Aalst ethical review board approved the study protocol.

Inclusion criteria were age older than 18 years, diagnosis of sepsis or septic shock as defined by the Sepsis‐III criteria,^29^ and admission from the emergency department (ED) within 24 h of hospital arrival, as it is reasonable to assume the continuation of their regular chronic medication until shortly before admission. Pregnant patients and patients receiving chronic renal replacement therapy (RRT) were excluded. We collected all specified data with regard to the ED visit and for the first 7 days of ICU stay, or until ICU discharge, whichever was sooner. Day 1 was defined as time of ED arrival until first morning (7 am) on the ICU ward. Mortality follow‐up was performed up to September 2021, with all patients completing at least 1 year follow‐up period (mean follow‐up for survivors 3,3 years).

### 2.2 Subgroups and metformin use

The study cohort was stratified according to metformin use and diabetes status. Stratified by metformin use, we distinguished two groups: diabetic patients using metformin (MET) and nonusers. The nonuser group was further subdivided into two subgroups: diabetic patients not using metformin (NOMET) and patients without diabetes mellitus (NODIAB). The timing of the last dose of metformin prior to ICU admission was unknown; metformin was discontinued on ICU admission as part of usual care.

Since differences in lactate and lactate kinetics may only become apparent in patients with elevated lactate, we defined a subset of patients with elevated lactate on admission (>2 mmol/L). This in accordance with previous studies, also stratifying by or setting a minimal admission lactate.^23^, ^30^, ^31^ Another subset analysis was performed on patients who fulfilled the Sepsis 3‐criteria for septic shock on Day 1 (both lactate ≥2 mmol/L and vasopressor use).

### 2.3 Lactate measurements

We defined admission lactate (LAC_adm_) as the lactate measurement closest to time of ED arrival, with a 6 h maximum tolerance. Lactate at 24 h (LAC_24h_) was recorded if within a range of 4 h (20‐28 h post ED arrival).

With no universal agreement on what dynamic parameter to use, as various studies each use different measurements and time frames,^28^, ^32^, ^33^, ^34^ we chose to analyse time‐weighted average lactate (LAC_TW_), as defined by Nichol et al,^33^ and the change in lactate (LAC_Delta24h_) over 24 h.

**FIGURE 1:**
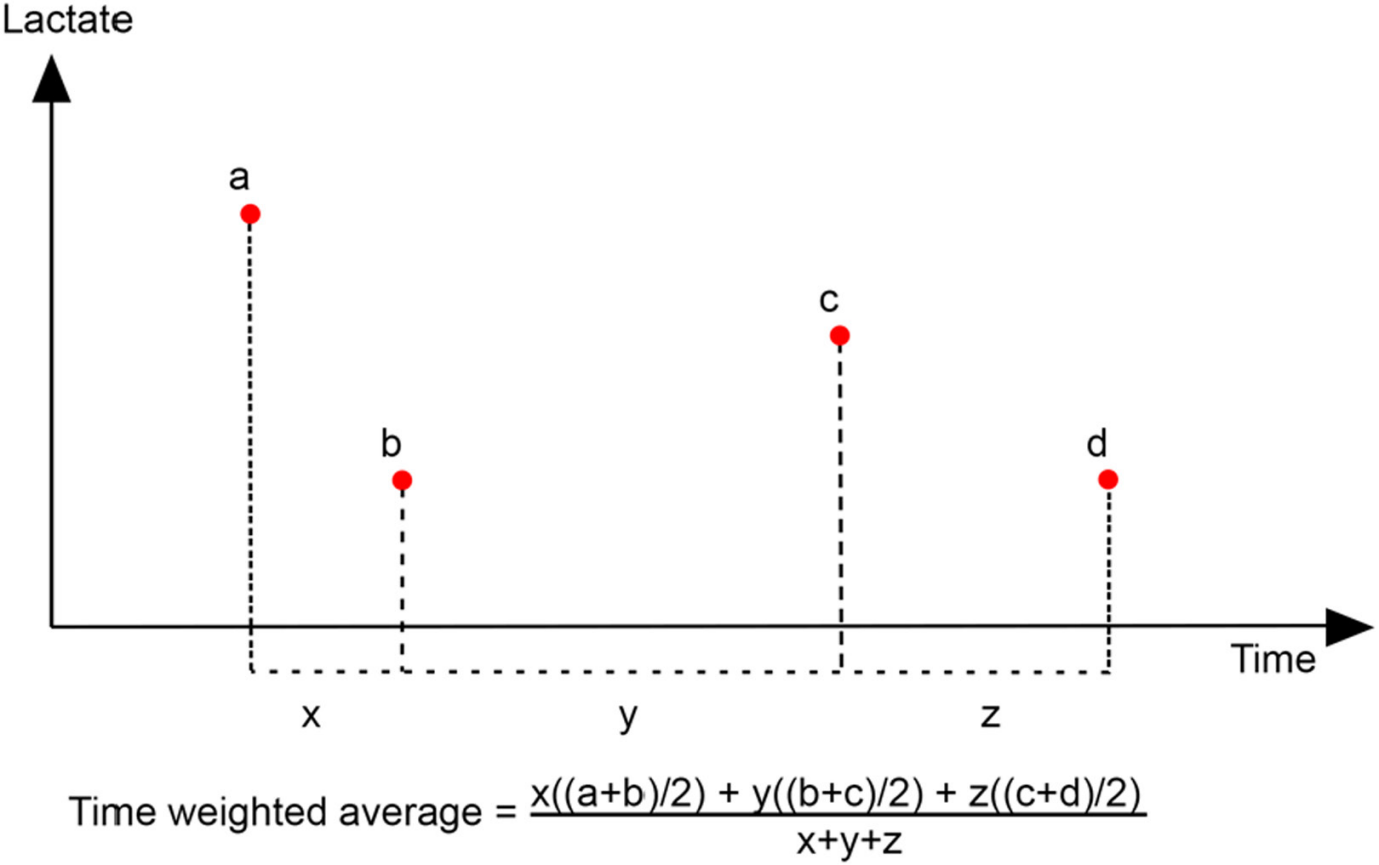
Diagram describing the calculation of time‐weighted average lactate (LAC_TW_), figure adapted from Nichol et al.^32^

Time‐weighted average lactate is an index of lactate homeostasis that is proportional to the amount of time spent at each concentration. We calculated this average concentration per day of ICU admission (LAC_TWdx_). It is calculated by first summing the median value between consecutive time points multiplied by the period of time in between them, and subsequently dividing it by the total time (see Figure 1). Although not yet often used, it has the advantage of taking into account the possible non‐linear and nonunidirectional evolution of plasma lactate levels, and can deal with differences in sampling frequencies and times, avoiding the potential effect of surveillance bias due to the increased blood lactate monitoring in more severely ill patients and patients with higher lactate levels.

The change in lactate over 24 h (LAC_Delta24h_) was calculated by subtracting the lactate on admission from the value at 24 h. LAC_Delta24h%_ describes the change in lactate over the first 24‐hour period as the percentage change from the admission blood lactate concentration.

As a blood lactate concentration reflects the balance between production and clearance, the term ‘lactate clearance’ was critiqued as being not physiologically correct to describe its evolution.^27^ We therefore choose to use the terms ‘dynamic lactate’, ‘lactate kinetics’ or ‘delta lactate’ to address the change of lactate over time.

### 2.4 Renal function

AKI was defined according to the Acute Kidney Injury Network (AKIN) stages,^35^ using both the serum creatinine and the diuresis criteria. In patients with unknown baseline serum creatinine and no known CKD, we used a back‐calculated estimated baseline serum creatinine based on the Modification of Diet in Renal Disease (MDRD) formula, in accordance with the Kidney Disease Improving Global Outcomes (KDIGO) guideline.^36^ As a sensitivity analysis, we also calculated AKIN based solely on admission creatinine value, as advised by the European Renal Best Practice position statement.^37^ We performed both analyses as the former assumes that there is a relatively low rate of CKD, while the latter assumes that AKI does not occur before hospitalization.

More detailed methodological information is published in the supplemental material (Appendix S1).

### 2.5 Statistical analysis

Continuous variables were expressed as median (25th–75th interquartile range) and compared using nonparametric Kruskal–Wallis (for three groups) and Mann–Whitney *U* test (for comparison between two groups e.g. survivors vs nonsurvivors) after testing for normality with histogram and QQ plots. Categorical variables were expressed as *n* (%) and compared using chi‐squared tests.

We applied a mixed‐effects model to analyse the repeated time‐weighted mean lactate measurements (LAC_TWdx_), because this model can handle the different follow‐up times, as patients died or were discharged from ICU at different time points, and it can to take into account the intra‐individual correlation (i.e. the fact that repeated measurements from a particular patient are correlated). We defined time‐weighted mean lactate as outcome parameter, with the individual patients as random effect, and day and group as fixed effects, adding all other parameters with significant difference between the groups. We chose as the most appropriate model the one with the smaller Schwarz Bayesian information criterion (BIC). Survival analyses were performed using the Kaplan–Meier survival curve and Log‐rank testing. All statistical analyses were performed using MATLAB Release 2020b (The MathWorks, Inc., Natick, Massachusetts, United States).

## 3 RESULTS

### 3.1 Population and baseline characteristics

The study included 467 septic patients, of which 125 (26.8%) with diabetes mellitus. Of those with diabetes, 77 (61.6%) used metformin (MET group; 34 (27.2%) in monotherapy and 43 (34.4%) in combination with other antidiabetic drugs) and 48 (38.4%) were not on metformin therapy (NOMET group).

Most of the baseline characteristics did not differ significantly between the groups (Table 1). The MET group was, however, significantly older than the NODIAB group and showed a higher BMI on admission compared with both the NOMET and the NODIAB group (median 28.7 kg/m^2^ vs. 25.17 kg/m^2^ and 24.87 kg/m^2^, respectively). Coronary or peripheral vascular disease (CADPVD) was significantly more common in both diabetic groups.

**TABLE 1:** Patients' baseline and clinical characteristics stratified by diabetes mellitus and metformin use

	MET (*n* = 77)	NOMET (*n* = 48)	NODIAB (*n* = 342)	*p*‐value
*Demographics*				
Gender (male), *n* (%)	50 (42,0%)	24 (50,0%)	202 (59,1%)	.423
Age, median (IQR)	75 (68–80)	71,5 (65–83)	70 (60–79)	.027^a^
BMI, median (IQR)	28,7 (26,1–33,5)	25,1 (23,0–29,9)	24,8 (22,2–28,4)	<.0001^b^
Year of Admission, median (IQR)	2017 (2015–2019)	2017 (2015–2019)	2017 (2015–2019)	.658
*Comorbidities*				
CADPVD, *n* (%)	29 (37,7%)	23 (47,9%)	79 (23,1%)	<.001^c^
CMP, *n* (%)	20 (26,0%)	15 (31,3%)	64 (18,7%)	.074
COPD, *n* (%)	32 (41,6%)	12 (25,0%)	117 (34,2%)	.163
Liver disease, *n* (%)	6 (7,8%)	3 (6,3%)	16 (4,7%)	.525
CKD, *n* (%)	17 (22,1%)	19 (39,6%)	33 (9,6%)	<.0001^d^
Malignancy, *n* (%)	11 (14,3%)	5 (10,4%)	63 (18,4%)	.305
Immunocompromised, *n* (%)	9 (11,7%)	8 (16,7%)	50 (14,7%)	.712
*Biochemical values*				
Admission CRP, median (IQR)	163,7 (71,5–310,0)	96,7 (40,2–303,7)	194,6 (70,6–346,6)	.050
Admission lactate, median (IQR)	2,8 (1,8–4,4)	2,7 (1,6–3,8)	2,3 (1,5–4,8)	.394
Baseline serum creatinine, median (IQR)	0,9 (0,7–1,2)^f^	0,9 (0,9–1,4)^f^	1,0 (0,8–1,2)^f^	.849
Number of baseline creats known	6	7	32	
Admission serum creatinine, median (IQR)	1,6 (1,2–2,4)	1,8 (1,2–2,9)	1,4 (0,9–2,5)	.020^a^
*Clinical and treatment information*				
Duration of ICU stay (days), median (IQR)	3,8 (2,5–5,8)	3,7 (2,4–5,6)	4,0 (2,5–6,8)	.556
SOFA score day 1, median (IQR)	7 (5–10)	6,5 (4–10)	7 (5–10)	.589
Maximal SOFA score, median (IQR)	7 (6–11)	8 (5–11)	8 (6–11)	.720
Bloodstream Infection, *n* (%)	23 (29,9%)	10 (20,8%)	114 (33,3%)	.206
Combination antibiotic therapy, *n* (%)	16 (20,8%)	10 (20,8%)	113 (33,%)	.038^e^
Use of aminoglycosides, *n* (%)	8 (10,4%)	4 (8,3%)	59 (17,3%)	.119
Antibiotic switch in first 48 h, *n* (%)	27 (35,1%)	14 (29,2%)	99 (28,9%)	.566
Mechanical ventilation, *n* (%)	30 (39,0%)	13 (27,1%)	139 (40,6%)	.197
Any vasopressor support, *n* (%)	40 (51,9%)	22 (45,8%)	191 (55,8%)	.390
Septic shock (day 1), *n* (%)	32 (41,6%)	15 (31,3%)	128 (37,4%)	.511
*Antidiabetic drug use*				
Insulin	12 (15,6%)	35 (72,9%)	–	<.0001
Sulfonyl urea	24 (31,2%)	17 (35,4%)	–	.623
DPP4 inhibitor	13 (16,9%)	4 (8,3%)	–	.175
SGLT2 inhibitor	1 (1,3%)	0 (0%)	–	.428
GLP1 receptor agonist	2 (2,6%)	0 (0%)	–	.260
Repaglinide	0 (0%)	1 (2,1%)	–	.203

Abbreviations: CADPVD, coronary artery disease/peripheral vascular disease; CKD, chronic kidney disease; CMP, cardiomyopathy; COPD, chronic obstructive pulmonary disease; MET, metformin users; NODIAB, nondiabetic patients; NOMET, diabetic non‐metformin users; SOFA, sequential organ failure assessment.

^a^ Significant difference (*p* < .05) between NOMET and NODIAB.

^b^ Significant difference between MET vs NOMET and MET vs NODIAB.

^c^ Significant difference between MET vs NODIAB and between NOMET vs NODIAB groups, no significance within diabetic patients (MET vs NOMET).

^d^ Significant difference between all groups.

^e^ Significant difference between MET vs NODIAB.

^f^ Baseline serum creatinine known in *n* = 32 NODIAB, *n* = 7 NOMET and *n* = 6 MET patients.

The prevalence of chronic kidney disease (CKD) differed significantly and was highest in NOMET (resp. 39.6%, 22.1% and 9.6% for NOMET, MET and NODIAB). Admission creatinine was not significantly different between metformin users and nonusers but was higher in NOMET than the nondiabetic controls. The NOMET group showed a tendency towards lower CRP on admission, without reaching statistical significance. With regard to antidiabetic drug use other than metformin, insulin use was higher in metformin nonusers. There was no significant difference in the use of other antidiabetic drug classes.

### 3.2 Clinical characteristics and severity of illness

In the overall population, 54.2% (*n* = 253) needed vasopressor support during their (first 7 days of) ICU stay, and 39% (*n* = 182) were mechanically ventilated. 37.5% (*n* = 175) of patients fulfilled the Sepsis 3‐criteria for septic shock at Day 1. The median SOFA score on Day 1 was 7 (IQR 5–10), the median maximal SOFA score during first week of ICU stay 8 (IQR 6–11). For all the aforementioned parameters, no significant between‐group differences were found.

Fewer metformin users were treated with a combination of antibiotics compared with nondiabetic patients. A numerically similar incidence of antibiotic combination therapy was seen in diabetic nonusers, but without reaching significance compared with NODIAB.

When divided into survivors and nonsurvivors based on ICU mortality, there was a significant association between mortality and higher SOFA score on Day 1, higher maximal SOFA score during ICU stay, admission lactate level, admission creatinine level, bloodstream infection, AKIN, mechanical ventilation and vasopressor use, among others. Similar results were seen when analysing 30 days mortality. (Tables S1 and S2.)

In the septic shock subset, severity of illness markers such as the median SOFA score on Day 1, maximal SOFA score during first week of ICU stay and need for mechanical ventilation, did not differ significantly between the groups. Overall median lactate on admission in the septic shock subgroup was 3.7 mmol/L, without significant between‐group differences.

### 3.3 Sepsis aetiology

Sepsis was caused by pulmonary infection in just over half of patients (51%, *n* = 238), with the second most prevalent source being urinary tract infection (16.7%, *n* = 78) (Table S3). The majority of cases were linked to bacterial infection (59.5%, *n* = 278). The causative micro‐organism remained unknown in 34.7% (*n* = 162) (Table S4). After merging smaller categories to meet the prerequisites for performing Chi2 testing, no significant between‐group differences regarding infection source or type of bacterial infection were revealed (Tables S3 and S5).

### 3.4 Effect of metformin use on lactate kinetics

Among metformin users, a significantly larger decrease in lactate levels was observed during the first 24 h, both in terms of absolute decrease (LAC_Delta24h_), and as percentage change compared with baseline (LAC_Delta24h%_) (Table 2). Time‐weighted mean lactate for day 1 (LAC_TWd1_) was not significantly different between groups.

**TABLE 2:** Lactate parameters

	MET	NOMET	NODIAB	*p*‐value
LAC_Admission_, median (IQR)	2,8 (1,8–4,4)	2,7 (1,6–3,8)	2,3 (1,5–4,8)	.394
LAC_Delta24h_, median (IQR)	‐1,5 (−0,5 ‐ ‐2,9)	‐1,1 (−0,5 ‐ ‐2,2)	−0,7 (−0,2 ‐ ‐2,2)	.047^a^
LAC_Delta24h%_, median (IQR)	−53,5 (−25,4 ‐ ‐69,4)	−42,7 (−26,4 ‐ ‐64,6)	−33,9 (−9,2 ‐ ‐60,4)	.010^a^
LAC_TWd1_, median (IQR)	2,0 (1,4–3,4)	1,7 (1,2–2,5)	1,8 (1,2–3,1)	.246

Abbreviations: LAC, _admission_ admission lactate; LAC_Delta24h_, difference in lactate at 24 h versus admission; LAC_Delta24h%_, difference in lactate at 24 h versus admission in percentage; LAC_TWd1_, Time‐weighted mean lactate for day 1; MET, metformin users; NODIAB, nondiabetic patients; NOMET, diabetic nonmetformin users.

^a^ Significant difference (*p* < .05) between MET group vs all other patients (NODIAB + NOMET), and between MET vs NODIAB.

Mixed‐effect model analysis of all patients did not show a significant correlation between daily time‐weighted mean lactate in between the three groups. When analysing only the subset of patients with elevated admission lactate ≥2 mmol/L, there was a significant difference between the groups with lowest values in MET (group estimate −0,43, 95% confidence interval [−0,77; −0,10], *p* = .012). Pairwise comparison showed lower values for metformin users vs nondiabetics (estimate −0,86, 95% confidence interval [−1,62; −0,09], *p* = .029).

### 3.5 Acute kidney injury and RRT

Incidence of AKI was high. The diuresis criteria, known to be the most sensitive, revealed the presence of AKI in 75% of the entire study population. Supplemental Table S6 presents the combined data from both creatine and diuresis criteria. There were no between‐group differences in incidence of any AKIN stage or moderate‐to‐severe AKIN (stage 2 or 3), nor in need for RRT during the study period or in start of RRT on the first day or first 2 days.

### 3.6 Survival analysis

Overall, most dichotomous mortality parameters (30d, 90d, 1 year mortality) did not differ significantly between groups (see Table S7 and Figure S1). Chi‐squared testing showed a statistically better ICU survival in the NOMET group (vs NODIAB), although this is not reproduced in other time frames and is likely due to small event size. In our extended follow‐up (>1 year), significantly higher survival rates were noted in both the metformin users and nondiabetics (see Figure S2). RRT‐free and AKIN‐free survival within the first 7 days showed no statistically significant between‐group differences (see Figure S3).

**FIGURE 2:**
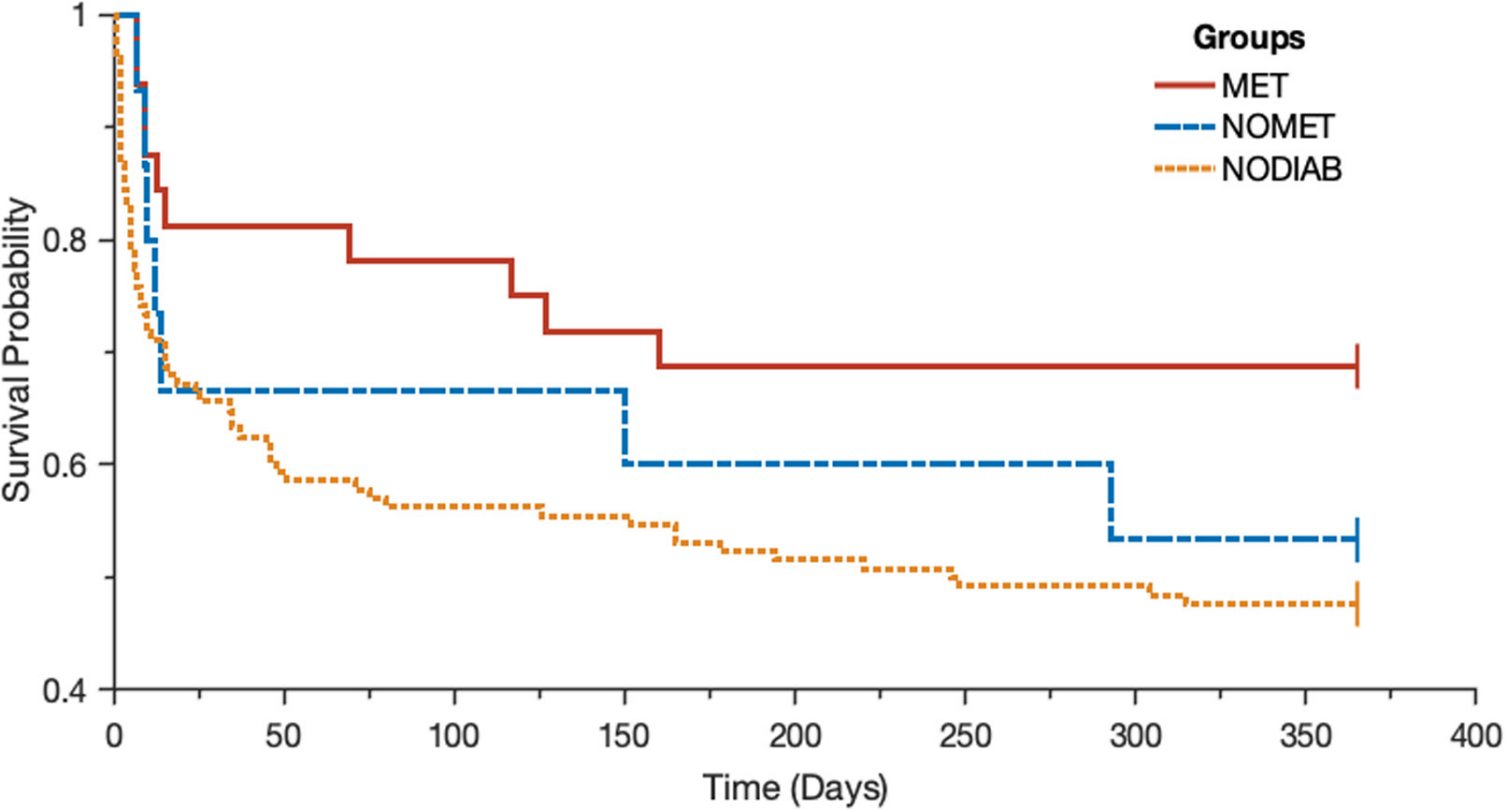
Survival probability for the septic shock subset (until 1‐year follow‐up). *MET* metformin users, *NOMET* diabetic nonmetformin users, *NODIAB* nondiabetic patients

In the septic shock subset, metformin users showed lower rates of ICU‐, 90 day‐ and 1 year mortality, when compared with the rest of the study population (Table S7). Survival data up to 1 year of follow‐up are shown in Figure 2. Logrank analysis showed a significant difference between MET and NODIAB in overall survival within the first 90 days and within the first year (resp. *p* = ,025, HR 0.42 (95% CI 0.23–0.76) and *p* = ,031, HR 0.49 (95% CI 0.29–0.84)). Other between‐group comparisons did not reach statistical significance.

### 3.7 DISCUSSION

Our study found no association between metformin exposure and initial lactate levels, thereby agreeing with several previous studies,^23^, ^25^, ^26^ and a recent review,^38^ although other authors found a positive association.^24^, ^30^, ^31^ Despite the similar admission values, metformin users with sepsis or septic shock showed a significantly larger decrease in lactate levels after admission, both over the first 24 h (delta lactate) and with regard to the mixed‐effect analysis of the first week of ICU stay (in patients with admission lactate ≥2 mmol/L). This is the first study to our knowledge to show this association, as the only previous study we found to publish data on lactate kinetics, by Park et al, found no difference in relative delta lactate over 6 h and 24 h.^25^ The difference in outcome can possibly be explained by the larger number of metformin users in this study, or by differences in study population, as for example our study had a larger proportion of patients with comorbidities, in need for mechanical ventilation (39 vs 7%, in metformin group), and mortality rates were almost twice as high (18% 30‐day mortality vs 10% 28‐day mortality, both for metformin group, respectively).^25^

Although the concept of lactate being an indicator of tissue hypoxia in shock states has been challenged, with lactate also potentially exhibiting positive effects serving as a fuel in these patients,^39^ decreases in lactate levels are almost universally associated with improved outcome.^17^, ^27^, ^39^ Therefore, this association between metformin use and higher decrease in lactate remains of clinical interest.

Importantly, this finding does not entail causality of any kind and may be accredited to several factors. Although it might be caused by a beneficial effect of metformin on inflammation, sepsis or septic shock resolution, in accordance with earlier basic sciences studies with mainly animal models,^8^, ^9^, ^10^, ^11^, ^12^, ^13^ there might be other confounding factors. Based on fundamental research, an impairment in lactate metabolism by metformin might be expected,^7^ potentially explaining the higher admission lactate levels found in some studies. Although our data on admission lactate do not support such association, the larger decrease in lactate might potentially be evoked by weaning of the impact of metformin on lactate metabolism. So, alternative to the proposed protective effect of metformin, metformin might have impacted and increased lactate on admission without the metformin users being equally severely ill. Very high levels of lactate on admission have previously been associated with good survival in MALA patients^40^ or septic metformin users.^23^ However, our data, in accordance with a recent meta‐analysis by Tan et al, showed a lower mortality despite showing no rise in initial lactate levels in metformin users.^38^ This does not exclude an impact of metformin on initial lactate levels, but even in our patients with septic shock and therefore a lactate >2 mmol/L, all severity of illness parameters were similar between groups, and lactates where only modestly elevated (median lactate on admission 3.7 mmol/L, no between‐group differences), making it less likely that patients needed ICU admission or mechanical ventilation or developed high SOFA scores solely because of this lactate.

Only a limited number of human studies specifically researched sepsis mortality stratified by premorbid metformin use. Several studies, with rather heterogenic inclusion criteria, reported lower mortality in metformin versus nonmetformin users,^23^, ^24^, ^30^, ^41^ while others failed to show any correlation.^25^, ^26^, ^42^ One study including both sepsis and septic shock patients only found an association in the septic shock subgroup,^24^ as is the case in our data. In 2019, two meta‐analyses were published, both showing a significantly lower mortality in metformin users vs nonusers (mixed 28d/30d/hospital mortality), strengthening the possibility of an association.^38^, ^43^ It must be mentioned that in the control group of these meta‐analyses, no differentiation was made between diabetics not taking metformin and nondiabetic patients, potentially masking confounding based on the diagnosis of diabetes. Tan et al mentioned the association was blunted when comparing metformin users with diabetic nonusers.^38^ Our data might suggest a similar trend, although the small event size in our group of diabetic nonmetformin users warrants prudency in its interpretation, but a more recent retrospective study by Yang et al did find lower mortality in metformin users versus diabetic patients not using metformin, using a larger sample size but older sample data, dating back to 2001–2012.^41^

It also remains unclear how metformin, lactate and mortality are interconnected. If metformin impacts both mortality and lactate levels, both can be due to separate pathways, explaining why for example the meta‐analysis of Park et al shows lower mortality despite showing no rise in initial lactate levels in metformin users,^25^ or lactate may be only one pathway of metformin's effect on mortality.

Our data did not provide evidence for any protective (or detrimental) effect of metformin on AKI or need for RRT. In the nonmetformin diabetic patients, incidence of CKD was higher, similar to several earlier studies.^24^, ^26^ Overall incidence of AKI was high in all groups, as expected in a critically ill population, combined with the effect of incorporating the diuresis criteria. We only found one previously published study including urinary output data, but this was limited to the first 24 h.^24^ Since then, definitions of AKI have evolved, and so our study is the first to use the more sensitive AKIN criteria.^35^ We did not find an association between metformin use and sepsis‐associated AKI but might have missed a potential impact on recuperation after sepsis‐associated AKI, as we did not have any follow‐up renal function data. As metformin has been shown to protect against progression to end‐stage renal disease in diabetic patients with CKD,^22^ it would be interesting for future studies to investigate if a protective effect also exists on progression of renal dysfunction because of sepsis‐induced AKI.

### 3.8 Strengths and limitations

Despite the prevalence of metformin use and sepsis worldwide, and the possible beneficial interaction between both derived from basic science data and animal studies, it has been the focus of little clinical research to date. The available literature draws varying conclusions, resulting in an overall low agreement. We therefore conducted this study to strengthen the available clinical evidence. Our study is unique in its long study period (up to 7 days of ICU stay), inclusion of all available lactate measurements over this period with analysis of both static and dynamic lactate parameters, availability of urinary output data and extensive mortality follow‐up.

This study also has several weaknesses that should be considered. First, the relatively small sample size, especially in the diabetic nonmetformin user subgroup, lowered the power of the analysis and made additional analyses comparing subgroups within each group impossible, for example analysis of the correlation between lactate and mortality according to metformin use and diabetes status.

Second, this is a retrospective analysis, with associated potential for confounding. Lacking randomization, we cannot differentiate whether any observed improvement is related to metformin itself or to confounding characteristics by which metformin users might have appeared to be more severely ill, and whether diabetic status influenced the likelihood of ICU admission.

For more definitive answers, further investigations including prospective studies, probably relying on substitute shock states such as cardiac surgery, are warranted to investigate this relationship.

Adding to this topic, most analysis did not control for potential confounders. Our groups were relatively well‐balanced in terms of baseline covariates, although the incidence of CKD was higher in the diabetics group and highest among diabetics not on metformin, similar to other studies regarding this topic,^24^, ^26^, ^31^ and CADPVD was higher in both diabetic groups. Age and BMI were higher among metformin users, but compared with the nondiabetic patients, this would all rather skew towards higher (instead of lower) mortality in the metformin group, as such undershooting any potential mortality benefit.

Third, we cannot guarantee all patients in the metformin user group really took metformin and had been taking it up until their admission. Our data were based on patient‐reported chronic medication use. We did not possess data on premorbid glycaemic control and glycaemic variability during ICU stay, although this might also impact mortality.^44^, ^45^

Fourth, lactate measurements were not routinely repeated in the first hours after arrival in the ED, making it impossible to analyse lactate kinetics over shorter time frames. This might have provided additional information as the systematic review of Vincent et al. showed that in critically ill patients, changes in blood lactate kinetics were clearly significant after 6 hrs in many studies and after 12 hrs in most.^27^

Lastly, as we conducted a single‐centred study in a large nonacademic ICU, generalization to other institutions may be inherently limited.

## CONCLUSION

In our data, preadmission metformin use is associated with a significantly larger decrease in lactate after admission in diabetic patients with sepsis or septic shock and with reduced mortality in septic shock. This is hypothesis‐generating as it strengthens the available data on the potential of an association between metformin use and decreased mortality, while also investigating its potential impact on lactate, which can act as a possible confounder or effector pathway. No evidence for any protective (or detrimental) effect of metformin on the kidney was found. More studies are needed to confirm our findings and unravel the complex interplay between metformin, lactate and sepsis, thereby exploring the potential use of metformin or its pathways in the treatment of sepsis.

## AUTHOR CONTRIBUTIONS

**Nina Van Moorter:** Conceptualization (equal); data curation (equal); formal analysis (lead); methodology (equal); software (lead); writing – original draft (lead); writing – review and editing (lead). **Thomas Tackaert:** Conceptualization (equal); data curation (lead); formal analysis (supporting); methodology (supporting); software (supporting); writing – original draft (supporting); writing – review and editing (supporting). **Koen De Decker:** Conceptualization (equal); data curation (supporting); methodology (supporting); writing – original draft (supporting). **Bruno Van Vlem:** Conceptualization (equal); data curation (supporting); methodology (equal); writing – original draft (supporting); writing – review and editing (supporting). **Nikolaas De Neve:** Conceptualization (equal); data curation (supporting); methodology (equal); writing – original draft (supporting); writing – review and editing (supporting).

## CONFLICT OF INTEREST

BVV reports consultancy agreements with Amgen, Astellas and Baxter. All other authors declare no conflicts of interest.

## Supporting information


Appendix S1

## Data Availability

The data that support the findings of this study are available from the corresponding author upon reasonable request.
