# Supplementary material for: New potential for an old kid on the block: Impact of premorbid metformin use on lactate kinetics, kidney injury and mortality in sepsis and septic shock, an observational study
Source: Endocrinol Diabetes Metab. 2022 Nov 28;6(1):e382. doi: 10.1002/edm2.382 (PMC9836235; doi:10.1002/edm2.382)

**Supplemental material**

**New potential for an old kid on the block: impact of premorbid metformin on lactate kinetics, kidney injury and mortality in sepsis and septic shock, an observational study.**

Nina Van Moorter^1^, Thomas Tackaert^2^, Koen De Decker^3^, Bruno Van Vlem^4^, Nikolaas De Neve^3^

Author affiliations:

^1^ Ghent University / Ghent University Hospital, Department of Internal Medicine, 9000 Ghent, Belgium,

^2^ Ghent University/ Ghent University Hospital, Department of Emergency Medicine, 9000 Ghent, Belgium,

^3^ OLVZ Aalst, Department of Anaesthesiology and Critical Care Medicine, 9300 Aalst, Belgium,

^4^ OLVZ Aalst, Department of Nephrology, 9300 Aalst, Belgium

**Appendices**

Appendix 1. Additional methodological information

**Additional tables**

Table S1 - Baseline and clinical characteristics for patients of the total cohort stratified by ICU mortality.

Table S2 - Baseline and clinical characteristics for patients of the total cohort stratified by 30d mortality.

Table S3 - Source of infection, stratified according to metformin use and diabetic status.

Table S4 - Microbiological etiology, stratified according to metformin use and diabetic status.

Table S5 - Microbiological etiology - simplified to larger categories for Chi squared testing.

Table S6­ - Incidence of acute kidney injury and need for renal replacement therapy, stratified according to metformin use and diabetic status.

Table S7 – Categorical survival analysis for patients in the total cohort and septic shock subset, stratified according to metformin use and diabetic status.

**Additional figures**

Figure S1 - Survival probability for the total cohort, stratified according to metformin use and diabetic status, 1 year follow-up time.

Figure S2 - Survival probability for the total cohort, stratified according to metformin use and diabetic status, entire follow-up time.

Figure S3 - Survival probability for RRT- and AKIN-free survival for the total cohort, stratified according to metformin use and diabetic status, first 7 days of ICU admission.

**Appendix 1. Additional methodological information**

*In- and exclusion criteria*

We included only those patients admitted to the ICU directly from the emergency department (ED) within 24h of hospital arrival, as it is reasonable to assume them to have taken their regular chronic medication until shortly before admission. A short stay on a tertiary location in between ED and ICU admission of less than 6h was allowed (operation room, regular hospital unit). Patients admitted in any hospital for a longer period of time before ICU admission were not included as they might have had their chronic therapy interrupted, inadvertently influencing any possible association.

*Diabetes mellitus*

Diabetes mellitus was defined by the use of anti-diabetic drugs. So-called diabetic patients treated only with diet were allocated to the non-diabetic group for the purpose of this study. We recorded all classes of preadmission chronic antidiabetic therapy, based on patient-reported medication use on ED or ICU admission, or any other recent patient encounters e.g. outpatient visits. One patient was excluded due to lack of information regarding chronic antidiabetic treatment.

*Measurements and characteristics*

All lactate values measured during ED and ICU stay were collected. Lactate measurements taken after the start of renal replacement therapy (RRT) were excluded from further analysis.

Urinary output was hourly measured as part of routine ICU care. We excluded data collected during large volume bladder irrigation for analysis, because of its interference with proper output measurements. Baseline serum creatinine was defined as the mean of all values obtained from the hospital’s database from 1 year up to 7 days prior to admission, with exclusion of those clearly corresponding to a transient episode of acute renal injury. In patients with unknown baseline serum creatinine and no known CKD, we used a back-calculated estimated baseline serum creatinine based on the Modification of Diet in Renal Disease (MDRD) formula, assuming an estimated glomerular filtration rate (eGFR) of 75 ml/min/1.73 m2. In this formula, we assumed all patients to be white, as most of our population is of European descent and race is not recorded in our EMR.

For survival analysis, we recorded date of death as well as ICU mortality, 30 day, 90 day and 1 year mortality. AKIN-free survival was defined as survival without reaching AKIN stage 2 or 3. RRT-free survival was defined as survival without need for acute renal replacement therapy (RRT).

Patients’ baseline characteristics, including age, height and weight, comorbidities, and admission CRP values were recorded. To account for changes in practice over the seven-year period of the study, the year of patient admission was included. Furthermore we included clinical information including source of infection, microbiological results, classes of antibiotic and antiviral therapy, corticoid use, vasopressor and inotrope use, use of mechanical ventilation, daily Sequential Organ Failure Assessment (SOFA) scores, need for RRT and days of RRT.

| **Supplemental table S1. Patients’ baseline and clinical characteristics stratified by ICU mortality** | | | |
| --- | --- | --- | --- |
|  | **Survivor** | **Nonsurvivor** | **P-value** |
| *Demographics* |  |  |  |
| Gender (male), n (%) | 232 (58,3%) | 33 (50,8%) | 0,256 |
| Age, median (IQR) | 72 (62-80) | 71 (62-79) | 0,770 |
| BMI, median (IQR) | 26 (23-29) | 25 (21-29) | 0,166 |
| Year of Admission, median (IQR) | 2017 (2015-2019) | 2017 (2015-2018) | 0,169 |
|  |  |  |  |
| *Comorbidities* |  |  |  |
| CADPVD, n (%) | 119 (29,9%) | 10 (15,4%) | 0,016 |
| CMP, n (%) | 89 (22,4%) | 9 (13,8%) | 0,119 |
| COPD, n (%) | 137 (34,4%) | 20 (30,8%) | 0,564 |
| Liver disease, n (%) | 20 (5,0%) | 5 (7,7%) | 0,378 |
| CKD, n (%) | 58 (14,6%) | 9 (13,8%) | 0,877 |
| Malignancy, n (%) | 65 (16,3%) | 13 (20,0%) | 0,464 |
| Immunocompromised, n (%) | 54 (13,6%) | 13 (20,0%) | 0,174 |
|  |  |  |  |
| *Biochemical values* |  |  |  |
| Admission CRP, median (IQR) | 180,6 (63,9-324,8) | 192,8 (81,4-356,5) | 0,351 |
| Admission lactate, median (IQR) | 2,3 (1,4-4,1) | 4,8 (2,3-9,1) | <0,0001 |
| Admission serum creatinine, median (IQR) | 1,4 (1,0-2,4) | 1,7 (1,1-3,0) | 0,031 |
|  |  |  |  |
| *Clinical and treatment information* |  |  |  |
| SOFA score day 1, median (IQR) | 7 (5-10) | 11 (7-14,25) | <0,0001 |
| Maximal SOFA score, median (IQR) | 8 (5-10,25) | 13 (10-16) | <0,0001 |
| Bloodstream Infection, n (%) | 118 (29,6%) | 28 (43,1%) | 0,031 |
| Combination antibiotic therapy, n (%) | 104 (26,1%) | 34 (52,3%) | <0,0001 |
| Use of aminoglycosides, n (%) | 42 (10,6%) | 29 (44,6%) | <0,0001 |
| Antibiotic switch in first 48h, n (%) | 119 (29,9%) | 21 (32,3%) | 0,695 |
| Mechanical ventilation, n (%) | 130 (32,7%) | 50 (76,9%) | <0,0001 |
| Any vasopressor support, n (%) | 199 (50,%) | 22 (45,8%) | <0,0001 |
| Septic shock (day 1), n (%) | 130 (32,7%) | 15 (31,3%) | <0,0001 |
| *MET* metformin users, *NOMET* diabetic non-metformin users, *NODIAB* non diabetic patients, *CADPVD* coronry artery disease/ peripheral vascular disease, *CMP* cardiomyopathy, *COPD* chronic obstructive pulmonary disease, *CKD* chronic kidney disease, *SOFA* sequential organ failure assessment. | | | |

| **Supplemental table S2. Patients’ baseline and clinical characteristics stratified by 30d mortality** | | | |
| --- | --- | --- | --- |
|  | **Survivor** | **Nonsurvivor** | **P-value** |
| *Demographics* |  |  |  |
| Gender (male), n (%) | 215 (58,3%) | 53 (54,1%) | 0,457 |
| Age, median (IQR) | 71 (61-79) | 74 (64-81) | 0,044 |
| BMI, median (IQR) | 26 (23-30) | 24 (21-28) | 0,010 |
| Year of Admission, median (IQR) | 2017 (2015-2019) | 2017 (2015-2019) | 0,777 |
|  |  |  |  |
| *Comorbidities* |  |  |  |
| CADPVD, n (%) | 102 (27,8%) | 27 (28,1%) | 0,948 |
| CMP, n (%) | 76 (20,7%) | 22 (22,9%) | 0,637 |
| COPD, n (%) | 127 (34,6%) | 30 (31,3%) | 0,536 |
| Liver disease, n (%) | 18 (4,9%) | 7 (7,3%) | 0,357 |
| CKD, n (%) | 52 (14,2%) | 15 (15,6%) | 0,718 |
| Malignancy, n (%) | 54 (14,7%) | 24 (25,0%) | 0,017 |
| Immunocompromised, n (%) | 47 (12,8%) | 20 (20,8%) | 0,048 |
|  |  |  |  |
| *Biochemical values* |  |  |  |
| Admission CRP, median (IQR) | 189,4 (66,8-330,2) | 163,7 (66,0-293,5) | 0,617 |
| Admission lactate, median (IQR) | 2,3 (1,5-4,1) | 3,7 (1,8-7,3) | <0,0001 |
| Admission serum creatinine, median (IQR) | 1,4 (1,0-2,4) | 1,7 (1,1-2,8) | 0,050 |
|  |  |  |  |
| *Clinical and treatment information* |  |  |  |
| SOFA score day 1, median (IQR) | 7 (5-10) | 9 (6-13) | <0,0001 |
| Maximal SOFA score, median (IQR) | 8 (5-10,75) | 11 (8-14) | <0,0001 |
| Bloodstream Infection, n (%) | 111 (30,2%) | 35 (36,5%) | 0,243 |
| Combination antibiotic therapy, n (%) | 100 (27,2%) | 38 (39,6%) | 0,02 |
| Use of aminoglycosides, n (%) | 43 (11,7%) | 28 (29,2%) | <0,0001 |
| Antibiotic switch in first 48h, n (%) | 117 (31,9%) | 23 (24,%) | 0,132 |
| Mechanical ventilation, n (%) | 124 (33,8%) | 56 (58,3%) | <0,0001 |
| Any vasopressor support, n (%) | 185 (50,4%) | 22 (45,8%) | <0,001 |
| Septic shock (day 1), n (%) | 120 (32,7%) | 15 (31,3%) | <0,0001 |
| *MET* metformin users, *NOMET* diabetic non-metformin users, *NODIAB* non diabetic patients, *CADPVD* coronary artery disease/ peripheral vascular disease, *CMP* cardiomyopathy, *COPD* chronic obstructive pulmonary disease, *CKD* chronic kidney disease, *SOFA* sequential organ failure assessment. | | | |

| **Supplemental Table S3. Source of infection.** | | | | | | | | | | |
| --- | --- | --- | --- | --- | --- | --- | --- | --- | --- | --- |
|  |  | **All** |  | **MET** | | **NOMET** | | **NODIAB** | | **P-value (Chi2)** |
| Pulmonary | | 238 (51,0%) | | 38 (49,4%) | | 24 (50,0%) | | 176 (51,5%) | | 0,70^†^ |
| Urinary | | 78 (16,7%) | | 16 (20,8%) | | 10 (20,8%) | | 52 (15,2%) | |  |
| Abdominal | | 45 (9,6%) | | 8 (10,4%) | | 4 (8,3%) | | 33 (9,6%) | |  |
| Skin and soft tissues | | 23 (4,9%) | | 6 (7,8%) | | 2 (4,2%) | | 15 (4,4%) | |  |
| Other | | 83 (17,8%) | | 2 (2,6%) | | 8 (16,7%) | | 66 (19,3%) | |  |
|  | Hepatobiliary |  | 27 (5,8%) |  | 2 (2,6%) |  | 3 (6,3%) |  | 22 (6,4%) |  |
|  | CNS |  | 15 (3,2%) |  | 1 (1,3%) |  | 0 (0,0%) |  | 14 (4,1%) |  |
|  | Orthopaedic |  | 6 (1,3%) |  | 0 (0,0%) |  | 2 (4,2%) |  | 4 (1,2%) |  |
|  | Other |  | 7 (1,5%) |  | 2 (2,6%) |  | 0 (0,0%) |  | 5 (1,5%) |  |
|  | Unknown |  | 28 (6,0%) |  | 4 (5,2%) |  | 3 (6,3%) |  | 21 (6,1%) |  |
| MET= metformin users, NOMET = diabetic non-metformin users, NODIAB = non diabetic patients. † Chi2 testing comparing all major infection source categories between NODIAB, NOMET and MET groups. | | | | | | | | | | |

| **Supplemental table S4. Microbiological etiology** | | | | |  |
| --- | --- | --- | --- | --- | --- |
|  |  | **All** | **MET** | **NOMET** | **NODIAB** |
| *A. Bacterial class † ‡* | |  |  |  |  |
| - Gram positive | |  |  |  |  |
|  | S. aureus, n (%) | 23 (4,9%) | 6 (7,8%) | 1 (2,1%) | 16 (4,7%) |
|  | CNS, n (%) | 4 (0,9%) | 1 (1,3%) | 0 (0,0%) | 3 (0,9%) |
|  | Streptococcus, n (%) | 69 (14,8%) | 9 (11,7%) | 2 (4,2%) | 58 (17,0%) |
|  | Other gram positive, n (%) | 1 (0,2%) | 0 (0,0%) | 0 (0,0%) | 1 (0,3%) |
| - Gram negative | |  |  |  |  |
|  | Enterobacterales, n (%) | 96 (20,6%) | 20 (26,0%) | 10 (20,8%) | 66 (19,3%) |
|  | Non-fermenters, n (%) | 15 (3,2%) | 1 (1,3%) | 2 (4,2%) | 12 (3,5%) |
|  | Gram neg. non-enterobacterales, n (%) | 22 (4,7%) | 3 (3,9%) | 0 (0,0%) | 19 (5,6%) |
| - Other | |  |  |  |  |
|  | Atypical, n (%) | 9 (1,9%) | 1 (1,3%) | 1 (2,1%) | 7 (2,0%) |
|  | Anaerobic, n (%) | 3 (0,6%) | 0 (0,0%) | 1 (2,1%) | 2 (0,6%) |
|  | Polymicrobial, n (%) | 36 (7,7%) | 4 (5,2%) | 1 (2,1%) | 31 (9,1%) |
|  |  |  |  |  |  |
| *B. Non-bacterial or mycobacterial † ‡* | |  |  |  |  |
|  | Viral, n (%) | 49 (10,5%) | 8 (10,4%) | 7 (14,6%) | 34 (9,9%) |
|  | Fungal, n (%) | 5 (1,1%) | 0 (0,0%) | 1 (2,1%) | 4 (1,2%) |
|  | Mycobacterial, n (%) | 1 (0,2%) | 0 (0,0%) | 0 (0,0%) | 1 (0,3%) |
|  |  |  |  |  |  |
| *C. Unknown etiology ‡* | | 162 (34,7%) | 29 (37,7%) | 25 (52,1%) | 108 (31,6%) |
| MET= metformin users, NOMET = diabetic non-metformin users, NODIAB = non diabetic patients. † A and B are not mutually exclusive: Bacterial and non-bacterial etiology could be simultaneously present in any one patient (eg. Influenza with pneumococcal infection). ‡ Percentages shown are within all patients. | | | | | |

| **Supplemental table S5. Microbiological etiology - simplified to larger categories for Chi squared testing.** | | | | | |
| --- | --- | --- | --- | --- | --- |
|  | **All** | **MET** | **NOMET** | **NODIAB** | **P-value (Chi2)** |
| S. aureus, n (%) | 23 (4,9%) | 6 (7,8%) | 1 (2,1%) | 16 (4,7%) | 0,21 |
| Streptococcus, n (%) | 69 (14,8%) | 9 (11,7%) | 2 (4,2%) | 58 (17,0%) |  |
| Enterobacterales, n (%) | 96 (20,6%) | 20 (26,0%) | 10 (20,8%) | 66 (19,3%) |  |
| Non-fermenters, n (%) | 15 (3,2%) | 1 (1,3%) | 2 (4,2%) | 12 (3,5%) |  |
| Polymicrobial or anaerobic, n (%) | 39 (8,4%) | 4 (5,2%) | 2 (4,2%) | 33 (9,6%) |  |
| Non bacterial, n (%) | 27 (5,8%) | 3 (3,9%) | 5 (10,4%) | 19 (5,6%) |  |
| Other or unknown, n (%) | 198 (42,4%) | 34 (44,2%) | 26 (54,2%) | 138 (40,4%) |  |
| MET= metformin users, NOMET = diabetic non-metformin users, NODIAB = non diabetic patients. | | | | | |

| **Supplemental table S6. incidence of AKIN^†^ and RRT** | | | | |
| --- | --- | --- | --- | --- |
|  | **MET** | **NOMET** | **NODIAB** | **P-value** |
| *Incidence* |  |  |  |  |
| Any AKIN | 60 (77,9%) | 41 (85,4%) | 271 (79,2%) | 0,559 |
| Any AKIN (est)^‡^ | 73 (94,8%) | 43 (89,6%) | 304 (88,9%) | 0,296 |
| AKIN 2 or 3 | 53 (68,8%) | 26 (54,2%) | 213 (62,3%) | 0,253 |
| AKIN 2 or 3 (est)^‡^ | 62 (80,5%) | 30 (62,5%) | 249 (72,8%) | 0,086 |
|  |  |  |  |  |
| *Time of occurrence* |  |  |  |  |
| Any AKIN on day 1 | 38 (49,4%) | 24 (50,0%) | 163 (47,7%) | 0,931 |
| Any AKIN on day 1 (est)^‡^ | 59 (76,6%) | 33 (68,8%) | 250 (73,1%) | 0,623 |
|  |  |  |  |  |
| *Acute RRT* |  |  |  |  |
| Any RRT needed | 6 (7,8%) | 3 (6,3%) | 23 (6,7%) | 0,931 |
| RRT on day 1 | 2 (2,6%) | 1 (2,1%) | 7 (2,0%) | 0,955 |
| RRT first 2 days | 6 (7,8%) | 2 (4,2%) | 14 (4,1%) | 0,377 |
| RRT = renal replacement therapy, MET= metformin users, NOMET = diabetic non-metformin users, NODIAB = non diabetic patients. † based on both creatinine and diuresis criteria. ‡ (est)= with use of calculated estimated baseline creatinine in case of unknown baseline. | | | | |

| **Supplemental table S7. Categorical survival analysis.** | | |  |  |  |
| --- | --- | --- | --- | --- | --- |
|  | **ALL** | **MET** | **NOMET** | **NODIAB** | **P-value** |
| *Overall population (n=467)* |  |  |  |  |  |
| Days until death or end of follow-up, n (IQR) | 606 (64-1.298) | 789 (91-1.389) | 454 (73-1.098) | 637 (48-1.302) | 0,498 |
| ICU mortality, n (%) | 66 (14,1%) | 6 (7,8%) | 3 (6,3%) | 57 (16,7%) | 0,033^†^ |
| 30-day mortality, n (%) | 98 (21,0%) | 14 (18,2%) | 10 (20,8%) | 74 (21,6%) | 0,797 |
| 90-day mortality, n (%) | 128 (27,4%) | 19 (24,7%) | 14 (29,2%) | 95 (27,8%) | 0,824 |
| 1-year mortality, n (%) | 172 (36,8%) | 26 (33,8%) | 20 (41,7%) | 126 (36,8%) | 0,673 |
|  |  |  |  |  |  |
| *Subset with septic shock (n=175)* | |  |  |  |  |
| Days until death or end of follow-up, n (IQR) | 397 (11-1.043) | 790 (122-1.368) | 386 (13-604) | 247 (8-1.001) | 0,113^‡^ |
| ICU mortality, n (%) | 45 (25,7%) | 3 (9,4%) | 2 (13,3%) | 40 (31,3%) | 0,021^§^ |
| 30-day mortality, n (%) | 55 (31,4%) | 6 (18,8%) | 5 (33,3%) | 44 (34,4%) | 0,231 |
| 90-day mortality, n (%) | 68 (38,9%) | 7 (21,9%) | 5 (33,3%) | 56 (43,8%) | 0,068^§^ |
| 1-year mortality, n (%) | 83 (47,4%) | 10 (31,3%) | 7 (46,7%) | 66 (51,6%) | 0,120^¶^ |
| MET= metformin users, NOMET = diabetic non-metformin users, NODIAB = non diabetic patients. † Significant difference (p<0,05) between NOMET and NODIAB. ^‡^ Significant difference (p<0,05) between MET vs rest. § Significant difference (p<0,05) between MET vs rest and NODIAB vs rest. ¶ Significant difference (p<0,05) between MET vs rest. | | | | | |

**Figure S1 - Survival probability for the total cohort, stratified according to metformin use and diabetic status, 1 year follow-up time.**

*MET* metformin users, *NOMET* diabetic non-metformin users, *NODIAB* non diabetic patients


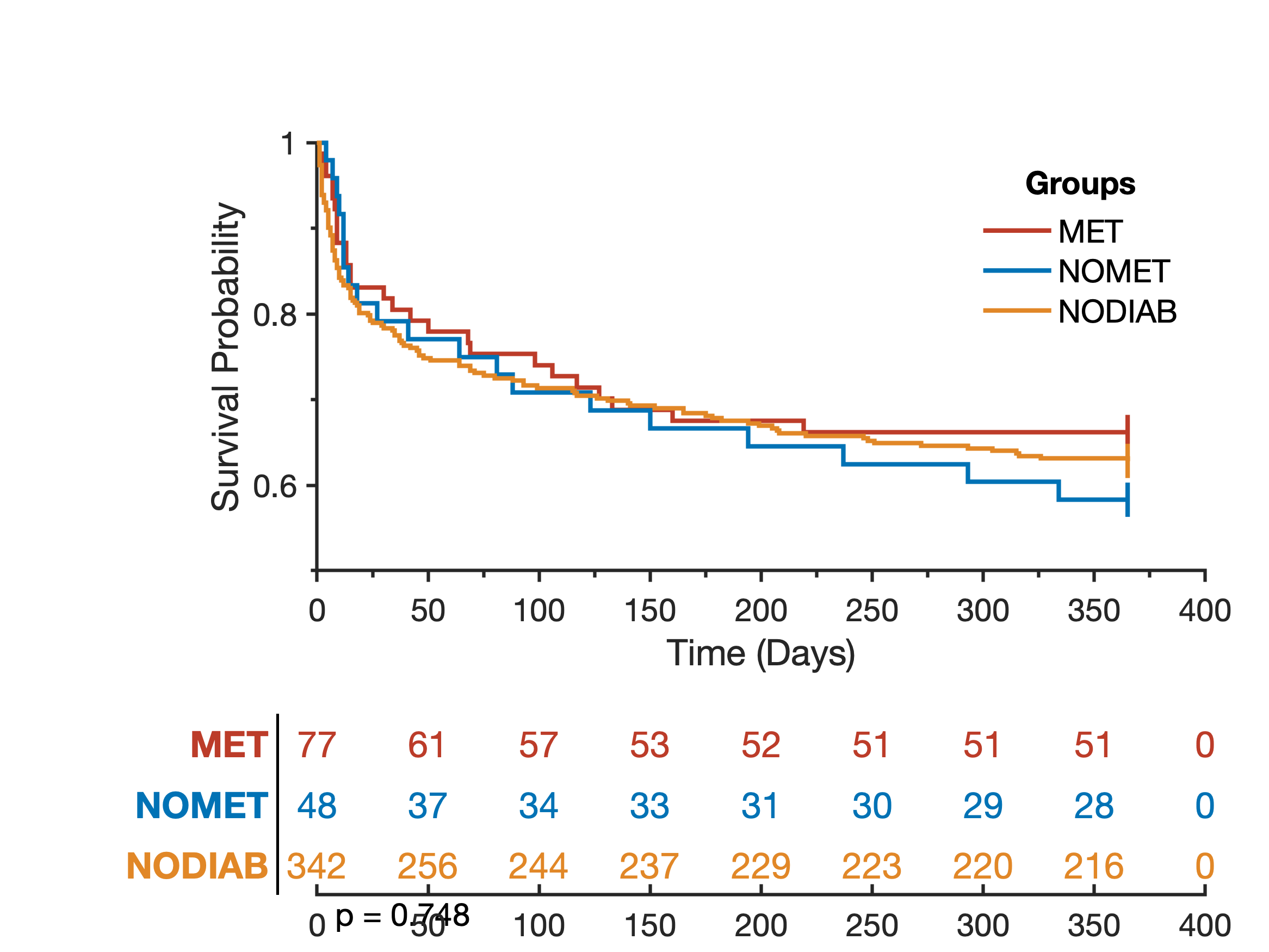


**Figure S2 - Survival probability for the total cohort, stratified according to metformin use and diabetic status, entire follow-up time.**

*MET* metformin users, *NOMET* diabetic non-metformin users, *NODIAB* non diabetic patients.


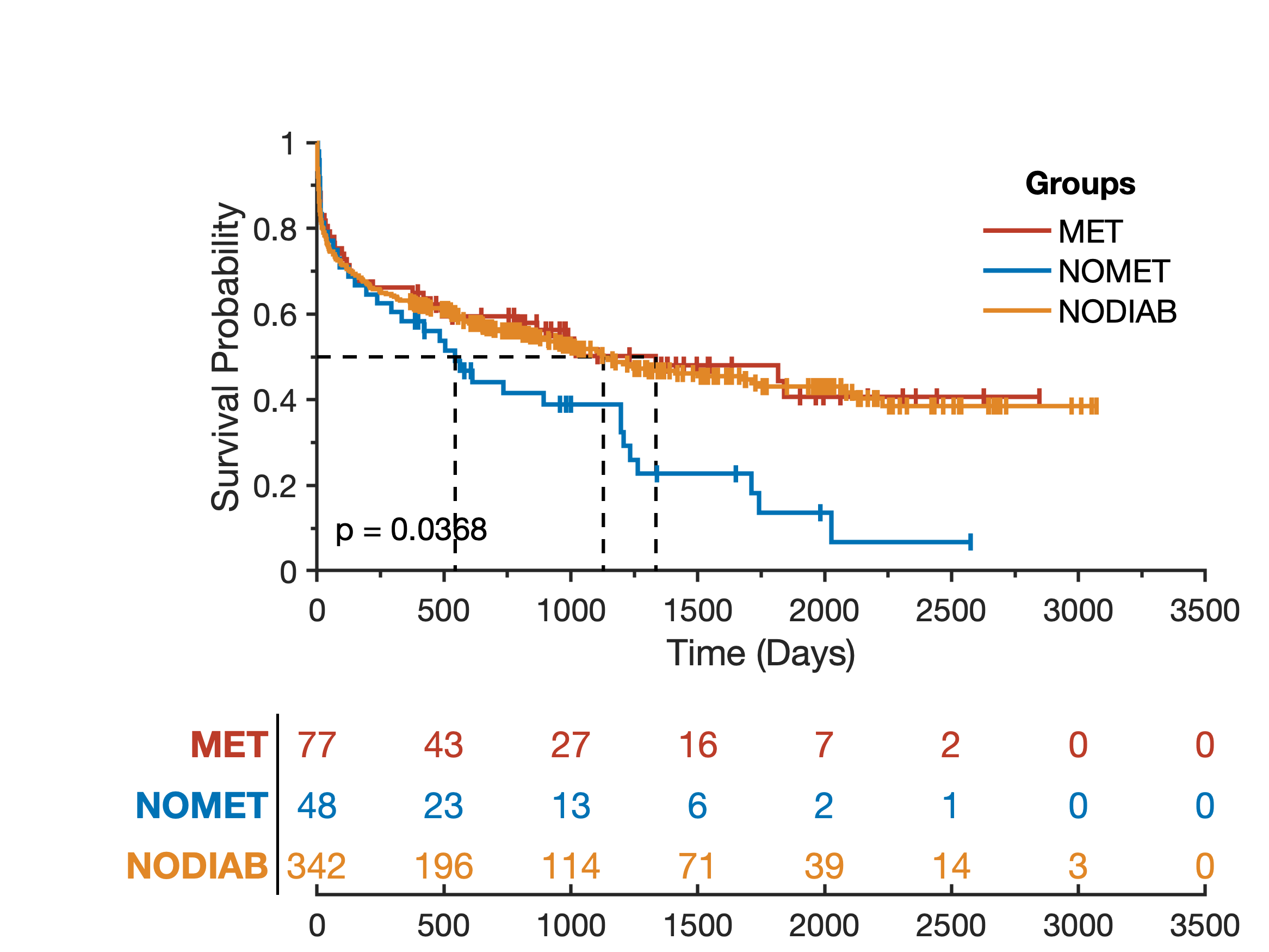


**Figure S3 - Survival probability for RRT- and AKIN-free survival for the total cohort, stratified according to metformin use and diabetic status, first 7 days of ICU admission.**


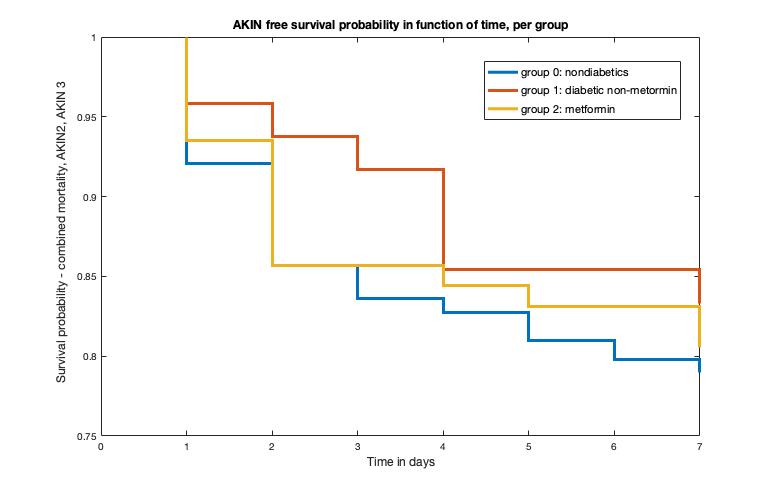

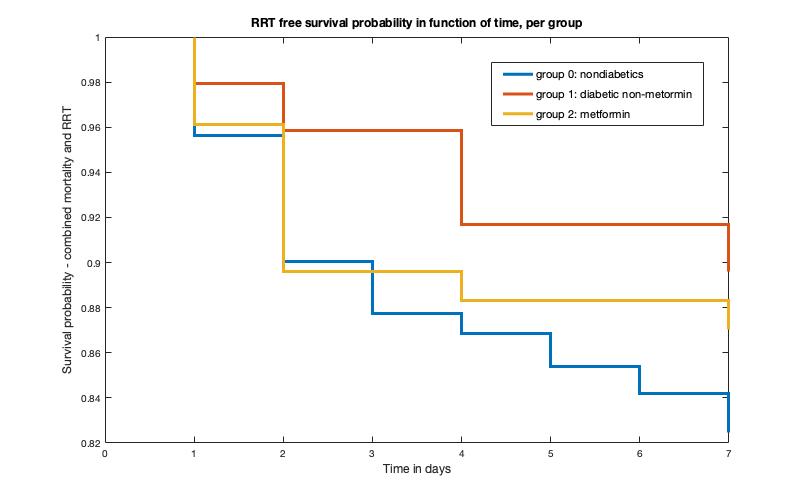

Supplement: Supplementary file 1 — Appendix S1 [file EDM2-6-e382-s001.docx]
